# Superfast Synthesis of Carbon Xerogels

**DOI:** 10.1021/acsomega.3c05824

**Published:** 2023-11-22

**Authors:** Abdurrahman Bilican, Priyanka Sharma, Nguyen Khang Tran, Claudia Weidenthaler, Wolfgang Schmidt

**Affiliations:** Department of Heterogeneous Catalysis, Max-Planck-Institut für Kohlenforschung, 45470 Mülheim an der Ruhr, Germany

## Abstract

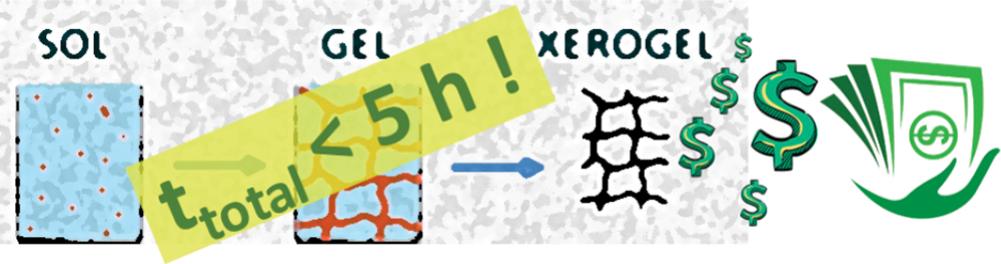

Carbon xerogels (CXs) provide unique opportunities for numerous applications in the areas of adsorption, separation, insulation, catalysis, and electrochemistry, but their use is hampered by time- and energy-intensive synthesis protocols. Synthesis protocols may require several days or more. Here, we report the synthesis of CXs requiring only 5 h using hydrothermal gelation and direct carbonization of the wet polymer. Drying of the polymer gel is of utmost importance for the synthesis of closely related carbon aerogels and is generally considered to be essential for the preparation of CXs. We show that skipping this step has no detrimental effect on the properties of CXs. They are identical to those obtained via conventional routes. With this “superfast” synthesis route, CXs with specific surface areas of about 700 m^2^ g^–1^ and total pore volumes up to 1.5 cm^3^ g^–1^ are obtained very time-efficiently and without loss of performance.

## Introduction

Carbon aerogels (CAs) and carbon xerogels (CXs) are unique carbon materials that possess not only exceptional porosity and specific surface area but also tunable pore volumes and pore sizes in the micro- and mesopore range. They are typically obtained by the carbonization of porous resorcinol-formaldehyde (RF) gels. The RF gels form via a sol-gel process. First, an RF polymer sol forms, and the polymer particles then connect to a three-dimensional RF gel with high porosity. Upon carbonization of dry RF gels at high temperatures in an inert atmosphere, the interconnected particles then transform into interconnected carbon particles, denoted here as primary particles. Due to their unique properties, CAs and CXs have obtained great attention in numerous fields that are decisive for the ecological transformation of the global economy. Their potential use was studied for electrochemical applications in fuel cells,^1,2^ supercapacitors^3−6^ or batteries,^7−9^ and for thermal insulation materials,^10^ as catalyst supports,^11,12^ and as highly efficient adsorbents.^13^ The high interest in these materials lies in the option to tune the material properties of meso- and microporous carbon for individual applications. The condensation of resorcinol and formaldehyde to an organic RF gel, as proposed by *Pekala*, provides the opportunity to tailor the pore and primary particle sizes of a porous polymer by the variation of pH and dilution of the reaction solution.^14^ The RF gel can be either converted into CAs or CXs via pyrolysis.^15^ RF gels form in a wet-chemical sol–gel process. Thus, the solvent is still present in the RF gel pores. Thermal removal of the liquid phase causes a high tension at the interface of the liquid and solid phases (capillary forces). The strong tension causes shrinkage and sometimes even a total collapse of the pore structure of the gel. Preventing the shrinkage of the pores is possible when drying with supercritical CO_2_ as the high surface tension at the liquid–solid interface is largely reduced. In such a way, CAs are obtained consisting mainly of pores and being the materials with the lowest known specific density (<0.0002 g cm^–3^).^16^ However, supercritical drying is rather time-consuming, and the synthesis of CAs is accordingly time-intensive. Even though CAs are among the materials with the highest known pore volumes and specific surface areas, the time- and labor-intensive synthesis prevents a wider application of CAs. An alternative to them is CXs, where the RF gel is dried thermally.^17−19^ Even though the porous structure of the gels deteriorates, CXs still possess extraordinary porous properties. They are thus promising candidates for a large number of applications.^9^ The reduction of pore sizes and porosity upon drying can be counterbalanced by the adjustment of synthesis parameters or by using additives.^20,21^ Therefore, CXs present a greater potential for commercial applications than their CA counterparts. Nonetheless, the commercial use of CXs is still challenged by extended synthesis protocols.^22^ To overcome this challenge, Wiener et al. accelerated the synthesis of RF gels using resorcinol-to-base catalyst ratios (RC values) between 1000 and 3000 and reported synthesis protocols of several days down to 24 h by heating the reaction mixture directly to 90 °C. Slightly smaller primary particles were obtained by the faster reaction at elevated temperatures. However, this was counterbalanced by an adjustment of the RC value.^23^ Alternatively, a hydrothermal sol–gel synthesis at elevated temperatures allows for the synthesis of the RF gel within hours.^24−26^ Cho et al. showed that RF gels can be obtained hydrothermally at 100 °C within 6 h.^24^ Further approaches to facilitate the carbon xerogel synthesis addressed the ambient drying process. A solvent exchange of the aqueous solution with a solvent with a lower surface tension is one way to reduce the capillary forces within the pores, thus reducing shrinkage of the organic gel during ambient drying. Slow heating of the RF gel at slightly elevated temperatures is also an option for gently removing the aqueous phase. Lin and Ritter showed that organic gels can be dried in a tube furnace under N_2_ flow first for 5 h at 65 °C and then for another 5 h at 110 °C without high losses of porosity.^27^ Léonard et al. dried RF gels without preliminary treatment via convective drying and then transformed them into CXs with SSA of 630 m^2^ g^–1^and pore volumes up to 1.25 cm^3^ g^–1^.^19^ Upon convective air-drying of organic xerogels at 70 °C, about 90% of the solvent evaporated after 1 h. The time for the remaining solvent to desorb depends on the pore size but does not exceed 8 h.^17^ Using microwave heating leads to even faster drying of the wet gels within 30 min.^28^ The microwave technique was then used to additionally accelerate the gelation and aging of the RF gel.^29,30^ Calvo et al. synthesized organic gels within 5 h. The resulting RF gels were comparable to those synthesized with conventional synthesis protocols requiring several days.^31^ The microwave synthesis was optimized, and the overall synthesis time (gelation, aging, and drying) of the RF gels was cut down to 3 h.^32^ Thus, major contributions were already made to reduce the cost and synthesis time for CXs. Still, further reduction of the synthesis time and simplifying the whole process are highly desirable.

Here, we report a convenient synthesis method that allows the synthesis of carbon xerogels within only a 5 h total synthesis time. Conventional heating is applied, and no other substances than described in the original report of *Pekala* are used; also no additional treatment of the RF gel is required after the gelation. The CXs obtained have SSAs of 600–700 m^2^ g^–1^ and pore volumes of 0.6–1.5 cm^3^ g^–1^. The major differences from the synthesis reported by *Pekala* are a more time-efficient preparation of the RF gel at temperatures of 80–120 °C and the omission of an independent drying step for the RF gels.

## Results and Discussion

Three synthesis routes for CXs were investigated in which only the molar ratio between resorcinol and the sodium carbonate catalyst, i.e., the RC ratio (see eq S1 in the Supporting Information), was varied as 500, 750, and 1000. The mass fraction, *M*%, was kept constant at 30% (see eq S2). These parameters were applied to three different synthesis routes, namely, the superfast (SF) synthesis, the hydrothermal (HT) synthesis, and the conventional (Conv) synthesis. For all synthesis routes, the wet RF gels were crushed prior to further processing, and carbonization was performed for 2 h at 1000 °C in all cases. Details of the synthesis procedures and characterization methods are reported in the Supporting Information.

The synthesis that we reported here for the first time allowed the synthesis of CX within only 5 h. On that 'SF*'* route, the RF gel was obtained after 1 h of hydrothermal reaction, and the wet RF gel was then directly carbonized. The CXs from the SF route are denoted as CX RC SF, with RC being the resorcinol-to-catalyst ratio. For comparison, reference materials were synthesized via two known routes. On the 'HT*'* route, the RF gel was synthesized hydrothermally as for the SF route, but then the RF gel was conventionally dried at 80 °C for 24 h prior to carbonization. The materials are denoted as CX RC HT, and their synthesis required 29 h in total. Finally, CXs were prepared via a 'Conv' route in which the gelation of the RF gel proceeded at 80 °C for 24 h, followed by drying of the RF gel at 80 °C for 24 h, and finally carbonization of the dry RF gel. CXs from this route are denoted as CX RC Conv, and their synthesis required 52 h in total.

**Figure 1:**
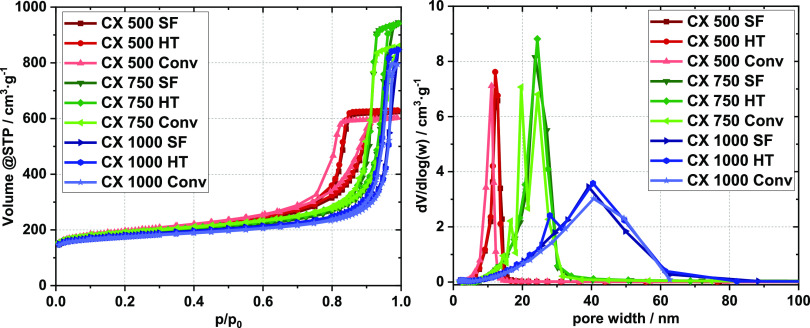
N_2_ sorption isotherms (left) of differently synthesized CX samples and the corresponding BJH pore size distributions (right).

The nitrogen sorption isotherms (Figure 1) show that all CXs synthesized in this study are micro- and mesoporous.^33^ In accordance with previous reports,^27,34^ the isotherms indicate significant changes of the pore system with variation of the RC value. The total pore volumes change significantly with the RC value (Table 1) and reach a maximum in the CX 750 series. Within the series, the total pore volumes, *V*_pore_, show no significant deviations for the materials from the SF and HT routes, whereas the materials from the Conv synthesis route always show somewhat lower total pore volumes. Apparently, neither the rapid polycondensation under HT conditions nor skipping of the extended drying of the RF gel causes a significant reduction of the total pore volume of the resulting CXs. During the initial pyrolysis of the wet RF gel, the major fraction of water desorbs rapidly up to a temperature of about 120 °C (Figure S1). The micropore volumes, *V*_mic_, increase moderately with increasing RC, which coincides well with the report of Rey-Raap et al., where a moderate increase of the micropore volume was observed when the pH of the reaction solution was reduced from 7 to 6.^35^ Moreno et al. showed that the carbonization conditions are crucial for the formation of micropores.^15^ However, within a given series with an identical RC value, the micropore volumes of the resulting CXs are identical for all routes. The formation of micropores thus seems to proceed alike. Direct heating of the wet RF gel to the carbonization temperature did not significantly alter the micropore volumes. Burn-off effects, as occurring in activation processes, may go along with an increase of the micropore volume.^13,36,37^ No such behavior is observed for the SF synthesis. In the mesopore region, increasing RC values induces increasing pore sizes (Figure 1). However, the CX 500 series shows an H2 hysteresis type, indicating ink bottle pores, the hysteresis shape shifts to an H1 type for the higher RC values, indicating more tubular pores.^38^ The pore size distributions do not change significantly for CXs obtained from different routes if they are synthesized with a given RC value.

**Table 1:** Specific Surface Area, *S*_BET_, External Specific Surface Area, *S*_ext_, Total Pore Volume, *V*_pore_, Micropore Volume, *V*_mic_, Mesopore Volume, *V*_mes_, and Pore Width, *d*_pore_, Derived from the N_2_ Sorption Isotherms of CX Samples

**sample**	***S***_**BET**_ **[m**^**2**^ **g**^**–1**^**]**	***S***_**ext**_^a^ **[m**^**2**^ **g**^**–1**^**]**	***V***_**pore**_^b^[cm^3^ g^–1^]	***V***_**mic**_^a^[cm^3^ g^–1^]	***V***_**mes**_^c^[cm^3^ g^–1^]	***d***_**pore**_^d^**[nm]**
CX 500 SF	692	279	0.968	0.166	0.802	12.8
CX 500 HT	710	286	0.975	0.170	0.805	12.1
CX 500 Conv	720	301	0.935	0.170	0.765	11.1
CX 750 SF	686	226	1.464	0.185	1.279	23.7
CX 750 HT	703	234	1.459	0.187	1.272	24.3
CX 750 Conv	718	232	1.332	0.193	1.139	24.4
CX 1000 SF	685	192	1.313	0.194	1.119	39.2
CX 1000 HT	675	193	1.312	0.189	1.123	40.4
CX 1000 Conv	665	181	1.231	0.190	1.041	40.6

a From *t*-plot.

b Total pore volume.

c *V*_mes_ = *V*_pore_ − *V*_mic_.

d Mesopore diameter from BJH.

**Figure 2:**
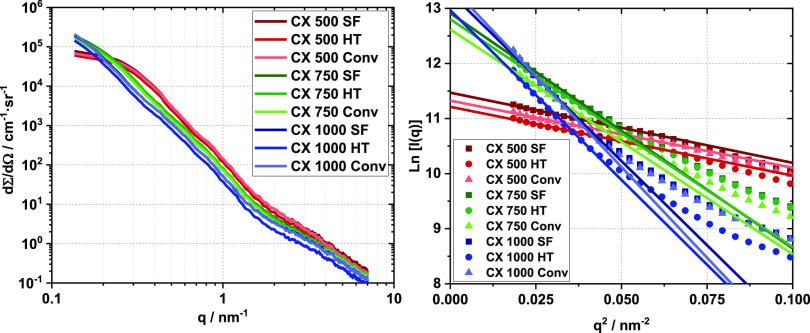
SAXS scattering curves (left) of differently synthesized CXs and the corresponding Guinier plots (right).

The data in Table 1 show that all apparent specific surface areas, *S*_BET_, vary around a mean value of 695 m^2^ g^–1^ ± 2.7%. This standard deviation is within the accuracy of the method, indicating quite similar apparent specific surface areas. The same holds for the micropore volumes, which vary around a mean value of 0.183 cm^3^ g^–1^ ± 6.0%. For fixed RC values, hardly any deviations of the external surface areas, *S*_ext_, are observed for carbon xerogels obtained via the three routes. With increasing the RC value, *S*_ext_ decreases for all routes, indicating larger primary carbon particle sizes. As the amount of base catalyst in the respective RC reaction mixtures is reduced, nucleation of particles is reduced as well and the RF particles grow larger, as also reflected by the increase of mesopore diameters with increasing RC values. SAXS curves for CXs synthesized with the same RC value show identical slopes, indicating their textural similarity (Figure 2).^39^ The presence of micropores in all of the CXs causes a shoulder in the *q* range of 2 to 7 nm^–1^.

The scattering intensities in the q region of 0.4 to 1 nm^–1^ reduce for CXs obtained with a higher RC value. As the scattering intensity in that region depends on the external surface area of the primary carbon particles, this observation is well in accord with the nitrogen sorption data. Calculation of the primary particle size via the Guinier approximation (eq S3) in the *q* range of 0.135 to 0.2 nm^–1^ resulted in the radii of gyration, *R*_G_, for the different CXs as reported in Table 2. From *R*_G_, the primary carbon particle sizes, *d*_part_, were calculated (eq S4). All primary particle sizes of CXs synthesized with the same RC value are identical within the experimental error irrespective of the synthesis route applied. Increasing RC values result in larger primary particle sizes, and this holds again for all synthesis routes. Furthermore, a significant shift of the plateau area toward smaller *q* values is observed with increasing RC value. The fitting ranges for the Guinier plots are not optimal for all samples due to the limited *q* range. Especially for the RC 750 and 1000 series, slightly overestimated particle diameters are likely the consequence. However, TEM images show that the general trend of increasing particle sizes is correct (Figure S3). Complementary nitrogen sorption and SAXS data thus provide a consistent picture of the textural properties of CXs. For fixed RC values, the textural properties of the CXs are reproducibly the same (Figure S2) irrespective of the synthesis route. Differences occur only if different RC values are used for the synthesis of the RF gels.

**Table 2:** *R*_G_ and *d*_part_ Were Obtained from SAXS Scattering Curves for Differently Synthesized CXs

**sample**	***R***_**G**_**[nm]**	***d***_**part**_^a^**[nm]**
CX 500 SF	6.2 ± 0.1	16.0 ± 0.2
CX 500 HT	6.1 ± 0.1	15.8 ± 0.3
CX 500 Conv	6.1 ± 0.1	15.6 ± 0.3
CX 750 SF	11.3 ± 0.1	29.2 ± 0.3
CX 750 HT	11.1 ± 0.1	28.8 ± 0.3
CX 750 Conv	11.1 ± 0.1	28.6 ± 0.2
CX 1000 SF	13.5 ± 0.1	35.0 ± 0.3
CX 1000 HT	13.6 ± 0.1	35.1 ± 0.3
CX 1000 Conv	14.1 ± 0.1	36.3 ± 0.3

a From eq S4 (supporting information).

Structural features of the CXs synthesized via different synthesis routes on the molecular level were studied by X-ray diffraction and Raman and X-ray photoelectron spectroscopies (XPS).

**Figure 3:**
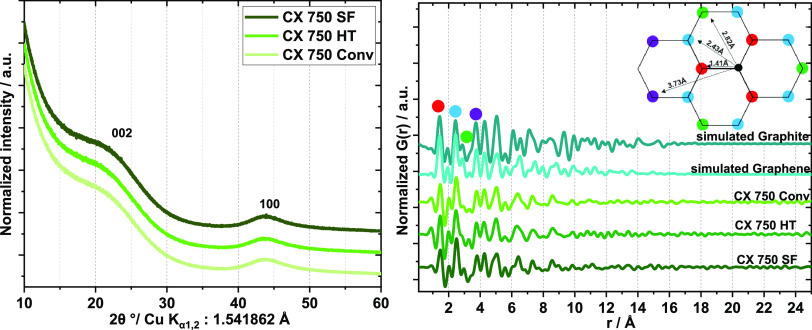
Diffraction patterns of differently synthesized CXs (left) and experimental PDFs of differently synthesized CXs and simulated PDFs of graphite and monolayer graphene (right).

Strongly broadened Bragg reflections indicate highly disordered graphitic carbon materials (Figures 3 and S4). The diffraction patterns show that the CXs obtained via the different synthesis routes show the same structural features. X-ray total scattering measurements and subsequent pair distribution function (PDF) analyses were performed, and the calculated PDFs are shown in Figures 3 and S5. The function indicates the probability of finding atom pairs with a certain distance. The first three peaks in the experimental PDFs of the CXs at around 1.41, 2.42, and 2.84 Å correspond to carbon–carbon distances in the carbon rings within graphene layers. All CXs display ordering similar to the simulated graphite and graphene structures in the first and second coordination spheres. Beyond these three peaks, the PDFs display noticeable differences originating from different packings of the graphene layers, different sizes and arrangements of the graphene units, and/or presence of defects. Since these xerogel materials are highly disordered and measured with laboratory Mo K_α_ radiation, the total scattering data are quite noisy, which manifest as ripples in the PDFs, which must not be confused with interatomic distances. The PDFs of the CXs synthesized via the different routes are very similar, indicating structural identity. They show no interatomic distances larger than 10 Å, indicating only short-range order within the graphene layers.

**Figure 4:**
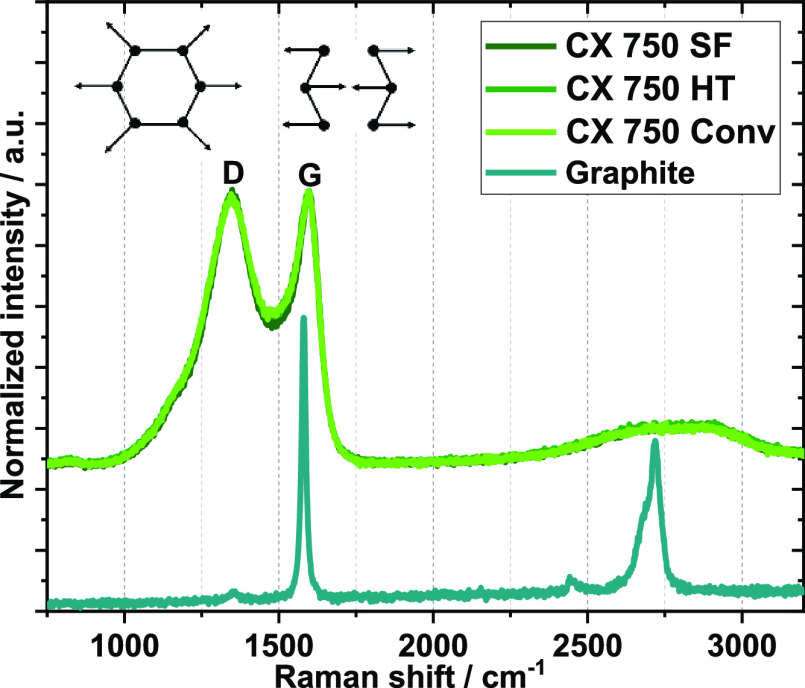
Raman spectra of differently synthesized CXs (overlapping, showing D1 bands at ∼1350 cm^–1^ and G bands at ∼1580 cm^–1^) and graphite as reference.

Figure 4 displays Raman spectra of the CX materials and a graphite reference material. CXs show the presence of bands at 1350 (D1) and 1580 cm^–1^ (G).^40^ The very broad nature of these bands confirms that the CX samples are highly defective, in agreement with the XRD and PDF data. The *A*_D1_/*A*_G_ ratio is the integrated intensity ratio of the D1 and G bands and is routinely used to quantify the degree of disorder in sp^2^ carbons. As can be seen in Table 3, all samples have a substantially higher degree of disorder than graphite, which again points toward poorly organized carbon. The spectra of the CXs obtained via the three routes are hardly discernible and again indicate the high degree of structural identity of the materials.

**Table 3:** Calculated Degree of Disorder for Differently Synthesized CX 750 and the Graphite Reference

**sample**	***A***_**D1**_**/*A***_**G**_
CX750/30SF	9 ± 1
CX750/30HT	10 ± 2
CX750/30Conv	11 ± 1
graphite	0.10 ± 0.01

Finally, the surface composition of the CXs was determined by XPS. Within the accuracy of the method, the elemental concentrations of the samples are identical (Table 4).^41,42^ The presence of Na in CX 750 Norm likely results from Na_2_CO_3_ used as the catalyst during synthesis. An understanding of the nature of surface carbon species can be obtained by calculation of the *D*-parameter.^43,44^ In the case of the graphite reference, considered as 100% sp^2^ carbon, this value is calculated as 23.5 eV. The *D*-parameters for the analyzed CXs are all alike and only slightly lower than that of graphite, implying similar sp^2^ carbon-rich surfaces after carbonization (Figure S8).

**Table 4:** Elemental Concentration and *D*-Parameter for Differently Synthesized CXs and Graphite As Obtained from XPS Data

**sample**	**surface composition**			***D*-parameter [eV]**
	**C at%**	**O at%**	**Na at%**	
CX750/30SF	∼98	∼2	n.d.	22.5
CX750/30HT	∼99	∼1	n.d.	22.5
CX750/30Conv	∼99	∼1	<1	22.5
graphite	>99	<1	n.d.	23.5

**Figure 5:**
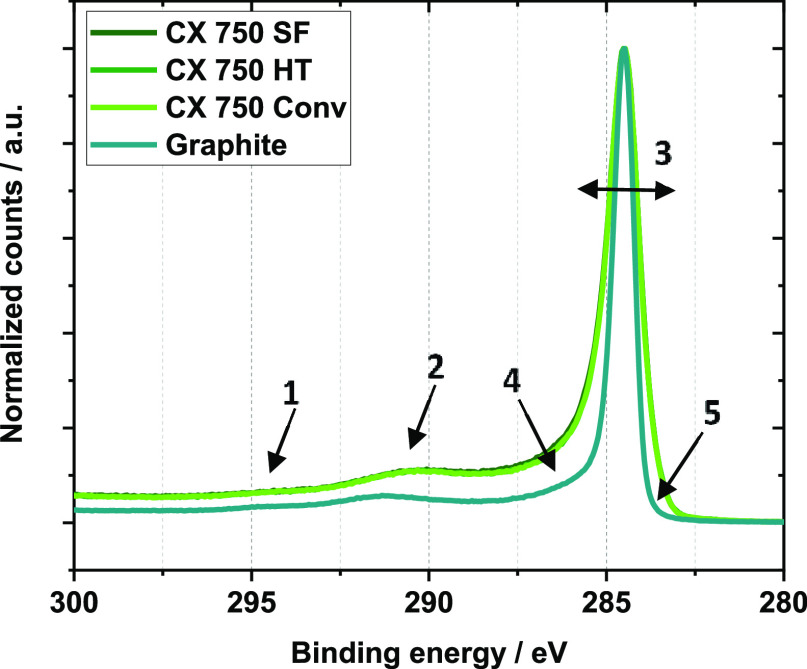
High-resolution XPS spectra in the carbon C 1s region of differently synthesized CXs (overlapping) and graphite.

High-resolution XPS spectra of the carbon C 1s region (Figure 5) again highlight the similarity between the CX 750 samples and their differences from graphite. Since the CXs from the three routes have identical structures, their spectra are overlapping and cannot be distinguished. However, clear differences in the C 1s spectrum of graphite are visible, marked by arrows in Figure 5. Total scattering data suggested short-range ordering within the graphene sheets of CXs. This agrees well with the report by Canal-Rodríguez et al., who show HRTEM images of xerogels consisting of interconnected graphene-like sheets without any order.^45^ Such lattice disruption seems to lead to changes in the π–π* shake-up features indicated by arrows 1 and 2 in Figure 5. The broadening of the main sp^2^ signal and its left shoulder (arrows 3 and 4) have been reported to arise from disordered carbon, whereas the presence of a shoulder at the right (arrow 5) might be due to point defects.^46^

Thus, variation of the RC value results in CXs with different properties, but for a given RC value, all three synthesis routes delivered identical materials. The wet RF gel can be directly carbonized without any detrimental burn-off effects. The overall structure of the carbon xerogels is highly disordered with the surface consisting mainly of sp^2^-rich carbon irrespective of the synthesis route applied. The porous properties of the carbon xerogels investigated here are mainly affected by the RC value applied during the gelation of the RF gel but not by the synthesis route. All routes allow the synthesis of CXs with pore volumes between 0.9 and 1.5 cm^3^ g^–1^ and specific surface areas between 600 and 700 m^2^ g^–1^.

## Conclusions

In summary, no significant differences are found for CXs synthesized via the SF route (<5 h) and those synthesized via the hydrothermal (29 h) or conventional (53 h) route. CXs can be synthesized within less than 5 h by using elevated temperatures for the gelation and direct carbonization of the wet RF gel. The porous properties as well as the molecular structure of the materials synthesized via different routes are indistinguishable. Drying during the initial stages of the pyrolysis step has the same effect as the extended drying process that is conventionally applied. Omitting this time- and energy-intensive drying of the RF gel has no detrimental effect on the final product. In combination with the rapid gelation of the RF gel via hydrothermal treatment, the synthesis route reported here allows the preparation of carbon xerogels in a highly efficient manner. This will stimulate further research and may result in the economic production of CXs for various applications.

## Supporting Information Available

Description of synthesis parameters and characterization methods, supplementary figures on TGA data, nitrogen and argon sorption isotherms, TEM and SEM images, SAXS data, PDF data, and XPS data (PDF)
