# Supplementary material for: Superfast Synthesis of Carbon Xerogels
Source: ACS Omega. 2023 Nov 22;8(48):45599–605. doi: 10.1021/acsomega.3c05824 (PMC10701873; doi:10.1021/acsomega.3c05824)
Supplement: Supplementary file 1 — ao3c05824_si_001.pdf [file ao3c05824_si_001.pdf]

Superfast Synthesis of Carbon Xerogels

Abdurrahman Bilican,[a] Priyanka Sharma,[a] Nguyen Khang Tran,[a] Claudia Weidenthaler,[a] Wolfgang Schmidt *,[a]

[a] *Max-Planck-Institut für Kohlenforschung, Department of Heterogeneous Catalysis,*
45470 Mülheim an der Ruhr, Germany

* corresponding author: schmidt@mpi-muelheim.mpg.de

SUPPLEMENTARY INFORMATION

Please note that additional references are given in the supporting information, which are not identical to those reported in the main document.

Synthesis

The molar ratio of resorcinol to formaldehyde, the RC value, for each synthesis is calculated according to eq. S1.

$$RC = \frac{n_{Resorcinol}}{n_{Na2CO3}} \tag{S1}$$

The second synthesis parameter, M%, (eq. S2), is the mass fraction of the mass of resorcinol and formaldehyde to the overall mass of the reaction solution and is equivalent to the dilution parameter which is often used in literature. In order to reduce the number of synthesis variables, M% was kept constant at a value of 30% in this study.

$$M\% = \frac{m_{Resorcinol} + m_{Formaldehyde}}{m_{total}} \tag{S2}$$

Superfast Synthesis of Carbon Xerogels

Resorcinol (ACS reagent ≥ 99 %, Sigma Aldrich) was dissolved in a 37 wt% aqueous formaldehyde solution (ACS reagent, 36.5-38.0%, stab. with 10-15% methanol) in a molar resorcinol/formaldehyde ratio (R/F) of 1:2. Then, an appropriate amount of water was added to achieve an M% of 30%. For the synthesis

of CXs with different porous properties, varying amounts of 0.05 M aqueous solution of Na_2CO_3 (Titripur ®, Merck KGaA Germany) was used to obtain the required RC values. The RC values were varied at 500, 750 and 1000. The solutions were shortly stirred and then poured into Teflon liners, which were inserted into steel autoclaves. The autoclaves were placed in a heating block at 120 °C for 60 min. The solidified RF gel was then crushed with a blade grinder to a powder with particle sizes in the millimeter range. The RF gel granules then were placed in a quartz glass tube that was heated for 2 h at 1000 °C in a Carbolite ® HST split tube furnace for carbonization (heating rate 10 °C.min^{-1}). The quartz glass tube was flushed continuously with N_2. The obtained carbon xerogels are denoted as CX RC SF, RC being the R/C ratio (eq. 1) and M% the mass percentage of the RF educts (eq. 2). The obtained carbon yield is comparable with the one of the carbonization of the dried gel (Supplementary Fig. S1).

Hydrothermal Synthesis of Carbon Xerogels

The RF gels were prepared as described for the superfast synthesis with RC variations of 500, 750 and 1000. The obtained gels were then crushed, dried at 80 °C for 24 h, and then placed in a quartz tube and carbonized in a Carbolite ® HST split tube furnace at 1000°C under N_2 flow (with a heating rate of 10 °C.min^{-1}). The nomenclature of these carbon xerogels is CX RC HT.

Conventional Synthesis of Carbon Xerogels

The reaction solutions for the normal synthesis were prepared as described above. After 10 min stirring, the solution was poured into glass vials, closed airtight and then placed for 24 h in an oven at 80°C for gelation. The solid RF gel was dried at 80 °C for 24 h and then carbonized under the same conditions as reported above. These carbon xerogels are denoted as CX RC Conv.

Characterization

Nitrogen Sorption

For N_2 sorption analyses at liquid nitrogen temperature, the carbon xerogels were degassed under vacuum for 12 h at 300 °C prior to the sorption measurements on a Micrometrics 3Flex instrument. For the data analysis, the MicroActive Software provided by Micromeritics was used. The Brunauer-Emmet-Teller (BET) theory was applied to calculate the specific surface area, S_{BET}.[S1] The evaluation of the BET surface area was performed under the Rouquerol criteria. [S2] As the materials investigated are not only mesoporous but also microporous, the BET algorithm does not provide correct specific surface areas but rather apparent specific surface areas. [S3] For calculation of the micropore volume, V_{mic}, and specific external surface area, S_{Ext}, the t-plot method was used applying the Harkins and Jura equation for calculating the thickness of the adsorbed layer within the capillary pores. [S4, S5] The pore width (mode), d_{pore}, in the meso- and macropore range was analyzed with the Barret-Joyner-Halendar (BJH) method using the desorption branches of the isotherms.

Small Angle X-ray Scattering (SAXS)

SAXS analysis was performed on a SAXSess instrument (Anton Paar) with slit-collimation of Cu K_α X-ray radiation. Carbon Xerogels were crushed to fine powders and then dried in a convection oven at 80 °C for 3 days. The powders were filled in a slit shaped mask with a width of 10 mm and thickness of 1 mm. The masks were sealed on both sides with transparent tesa® duct tape. The scattering curves were obtained in the q range of 0.13 – 7 nm^{-1} by binning of 10 frames measured with an exposure time of 60 s with a Dectris Mythen 1K strip detector with 10 mm widths. The scattering curves were desmeared with the SAXSquant (V.3.90.2743.35) software algorithm. Using a glassy carbon standard (NIST SRM 3600), the scattering curves were calibrated to an absolute scale according to the specification of NIST. [S6] Data evaluation was performed in the Guinier range to gain information on the primary particle dimensions using the Guinier approximation (eq. S3): [S7]

$$I(q) = I_0 \cdot exp\left(-\frac{R_G^2 q^2}{3}\right) \tag{S3}$$

Eq. S3 contains the parameter, R_G, the radius of gyration which is model independent and can be applied to any particle shape. [S8] For particles known to be spherical, the mean particle diameter, d_{part}, can be calculated with eq. S4. [S9]

$$d_{part} = 2 \cdot \sqrt{\frac{5}{3}} \cdot R_G \tag{S4}$$

Ex-situ X-ray Diffraction, Total Scattering and Pair Distribution Function (PDF) Analysis

Ex-situ X-ray diffraction data were recorded on a Rigaku SmartLab diffractometer equipped with a rotating anode (9 kW, 45 kV, 200 mA) in Bragg-Brentano geometry (Cu $K_{\alpha1,2}$: 1.541862 Å) for qualitative average measurements. Data were collected continuously in the range of 10 – 90° 2θ in steps of 0.01° and a scan speed of 0.5° min^{-1} with a HyPix-3000 multi-dimensional detector in 1D mode. For each sample, three scans were collected and summed after data collection. The samples were placed on background-free silicon sample holders. Total scattering experiments were performed on a Stoe STADI P transmission diffractometer (Mo $K_{\alpha1}$) for local structure analysis. The instrument is equipped with a curved Ge (111) monochromator and a Dectris Mythen 1K detector. The powder samples were prepared in borosilicate capillaries having a diameter of 0.7 mm. The program PDFgetX3 was used for processing pair distribution functions (PDFs) from the scattering data and PDFgui v1.1.2 was used to visualize the PDFs. [S10, S11] For correction of background scattering from air and sample container, an empty glass capillary was measured. PDFs were calculated for Q_{max} of 15 $Å^{-1}$. The experimental PDFs were compared with simulated ones for graphite and graphene monolayer. Crystallographic information file (CIF) for graphite (ICSD: 76767) was provided by the Inorganic Crystal Structure Database (ICSD, © FIZ Karlsruhe). The graphene monolayer structure was approximated by setting a very large interlayer spacing value of 500 Å to the graphite structure for eliminating interlayer pair correlations. Si NIST 640e standard data were used for obtaining the instrumental parameters, *i.e.*, Q_{damp} and Q_{broad} as 0.0071 $Å^{-1}$ and 0.0039 $Å^{-1}$, respectively.

Raman Spectroscopy

For determining the degree of disorder in carbon xerogels, Raman spectra were collected with Renishaw inVia Raman spectrometer using a 532 nm laser, collecting 5 acquisitions per point with an exposure time of 10 s and 1% of the total laser power in the range 100 – 3200 cm^{-1}. A total of 3 points per sample were measured to check the homogeneity of samples. Timcal Timrex® SFG150 synthetic graphite powder was measured as a reference. Prior to measurements, calibration was performed with a Si standard wafer. The spectra were then fitted in TOPAS software v5 with the model described by Sadezky. [S12]

X-ray Photoelectron Spectroscopy (XPS)

XPS was utilized to characterize the surface chemistry and nature of carbon species in the samples. The powder samples were filled in shallow Ti sample holders and measured on a SPECS spectrometer with a hemispherical analyzer (PHOIBOS 150 1D-DLD). The monochromatized Al K_α X-ray source (E = 1486.6eV) was operated at 15 kV and 200 W. Analyzer pass energies of 20 eV and 50 eV were applied for the high-resolution and survey scans, respectively. The pressure during the experiment in the analysis chamber was approximately 10 x 10^{-10} mbar. To minimize the effect of charging, a flood gun was switched on before measuring each sample. The data were analyzed using the CasaXPS software v2.3.26rev1.1S. [S13] All C 1s spectra were calibrated and normalized to the main photoelectron peak of sp^2 hybridized C at 284.5 eV. To determine the nature of surface carbon species (sp^2 / sp^3), the C KLL Auger regions were recorded with 100 eV pass energy, 0.5 eV step size for 50 scans. [S14] D-parameters were then calculated by differentiating C KLL Auger spectra using Savitsky-Golay Quadratic smoothing width of 9 and SP background type in CasaXPS.

Transmission Electron Microspcopy (TEM)

The carbon xerogels were measured on a Hitachi H-7100 Transmissiopn Electron Microscope equipped with tungsten cathode operating at 100kV. Images were acquired through a side entry CCD camera and processed with the MegaView III software package. For sample preparation, TEM grids were wiped along the inside walls of the glass vials containing the carbon xerogels. No solvent or adhesive was used.

Scanning Electron Microscopy (SEM)

For acquiring SEM images, a Hitachi TM3030 Tabletop Scanning Electron Microscope was used, operating at an acceleration voltage of 15kV. For sample preparation, the carbon xerogel powders were spread (dry) on conductive double sided adhesive carbon tapes which were then placed on the SEM sample holder.

Supplementary Figures and Tables

1. Thermogravimetric analysis (TGA)

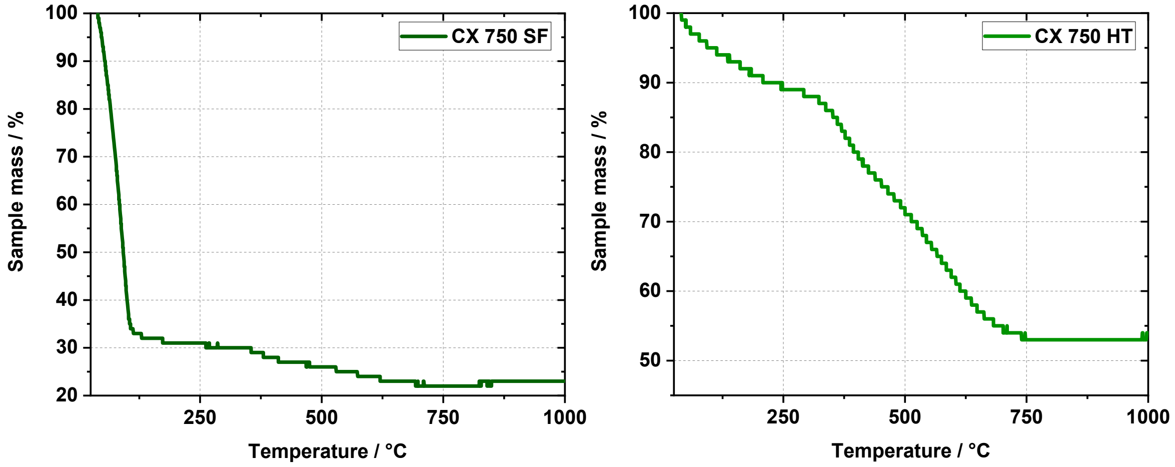

Fig. S1. Thermogravimetic analysis of the carbonisation of a wet (left) and dry gel (right) upon heating to 1000 °C with a heating rate of 5 K/min under Ar flow.

Fig. S1 shows the TG scan of CX 750 SF (carbonization of a wet gel) and CX 750 HT (carbonization of a dry gel) at 5 K/min up to 1000 °C under argon flow. Both samples release a fraction of volatile compounds up to a temperature of about 250°C, which is significantly higher for the CX 750 SF material as it has not been dried. At the temperature of 250°C, both samples are fully dried RF gels. From these dry RF gels, 74 wt% (23 / 31 = 0.74) of carbon is obtained for the RF gel from the CX 750 SF synthesis mixture after heating to 1000°C under argon, the RF gel from the CX 750 HT yields 61 wt% (53 / 87 = 0.74) of carbon.

2. Nitrogen sorption

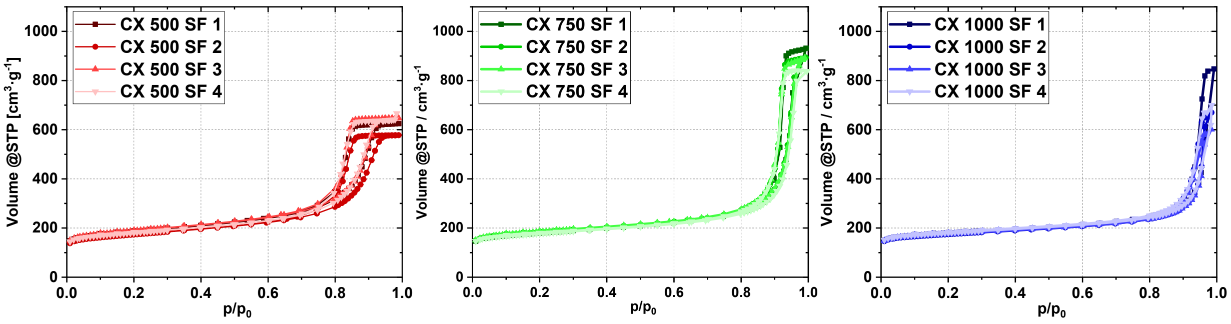

Fig. S2. N_2 isotherms of the reproduction series of SF samples with different RC values. The samples were synthesised under the same conditions four times.

Tab. S1. Specific surface area S_{BET}, external specific surface area S_{ext}, total pore volume V_{pore}, micropore volume V_{mic} , derived from the N_2 sorption isotherms of CX samples.

sample	S_{BET} [m²·g⁻¹]		S_{ext}^{a} [m²·g⁻¹]		V_{pore}^{b} [cm³·g⁻¹]		V_{mic}^{a} [cm³·g⁻¹]	
**CX 500 SF 1**	692	673±17	279	259±12	0.968	0.949±0.035	0.166	0.166±0.005
**CX 500 SF 2**	646		252		0.906		0.157	
**CX 500 SF 3**	677		248		0.926		0.171	
**CX 500 SF 4**	676		256		0.994		0.168	
**CX 750 SF 1**	686	685±8	226	230±5	1.464	1.383±0.058	0.185	0.182±0.003
**CX 750 SF 2**	689		232		1.386		0.181	
**CX 750 SF 3**	693		236		1.381		0.182	
**CX 750 SF 4**	671		224		1.299		0.178	
**CX 1000 SF 1**	685	680±9	192	193±3	1.313	1.092±0.139	0.194	0.191±0.003
**CX 1000 SF 2**	668		194		1.038		0.186	
**CX 1000 SF 3**	676		190		0.932		0.191	
**CX 1000 SF 4**	691		197		1.085		0.194	

a: from t-plot, b: total pore volume, c: $V_{mes} = V_{pore} - V_{mic}$, d: mesopore diameter from BJH

3. Transmission Electron Microscopy (TEM)

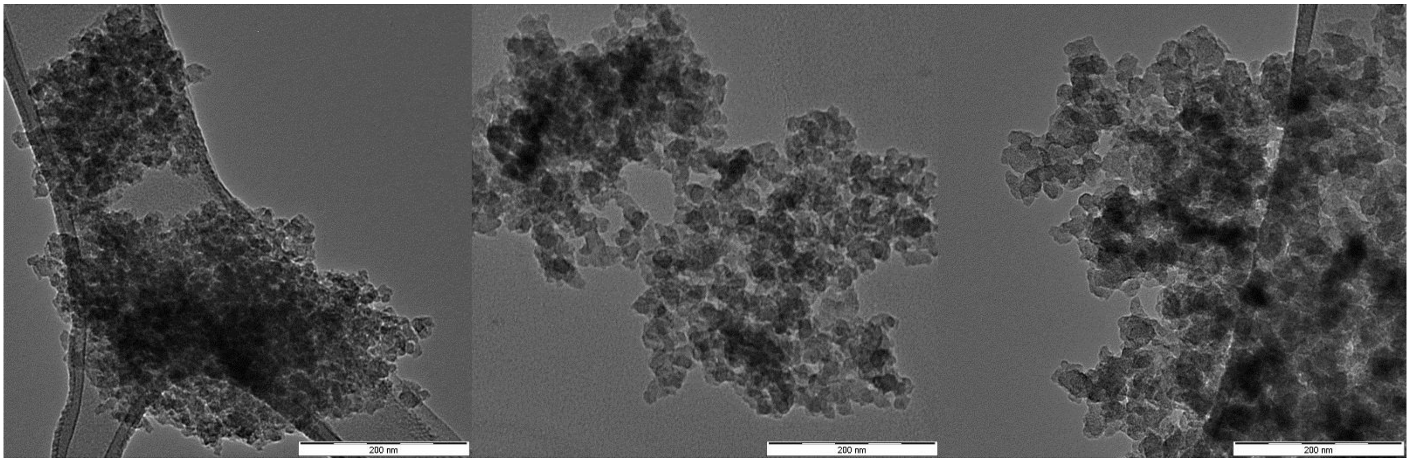

Fig. S3. TEM images of CX 500 SF (left), CX 750 SF (middle) and CX 100 SF (right). Scale bar 200 nm.

4. Total scattering data and pair distribution function analysis (PDF)

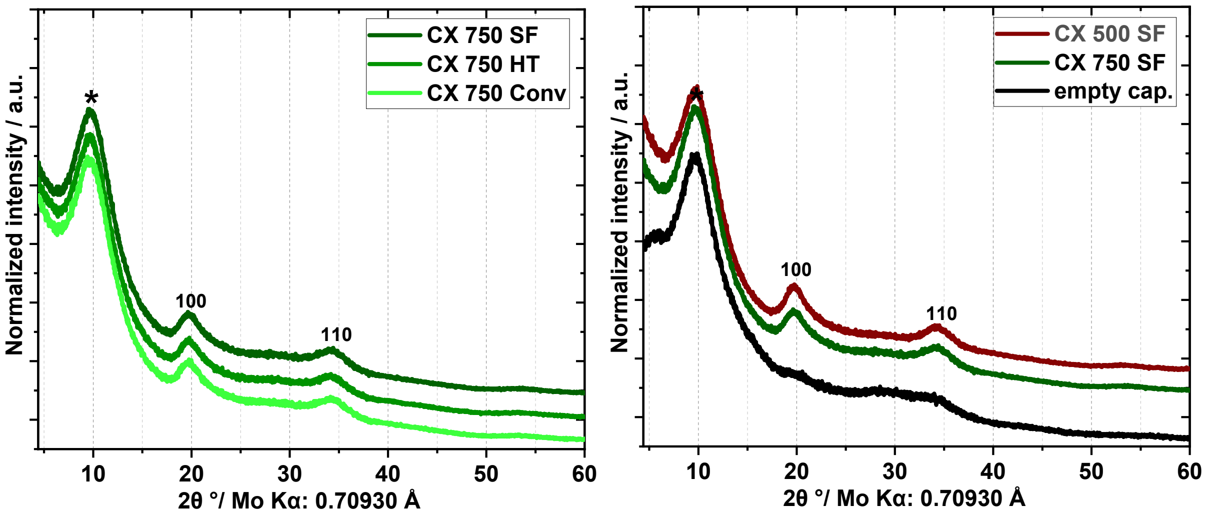

*Fig. S4. Diffraction patterns of differently synthesized CX gels. * scattering from glass capillary overlays the (002) reflection.*

The diffraction patterns for the samples reveal the high level of disorder as only very broad Bragg reflections are observed (Fig. S4). Additionally, the (002) reflection overlays with the diffraction originating from the capillary used for sample preparation for these measurements. However, the presence of broad reflections at approximately 20° and 34° 2θ indicates local ordering.

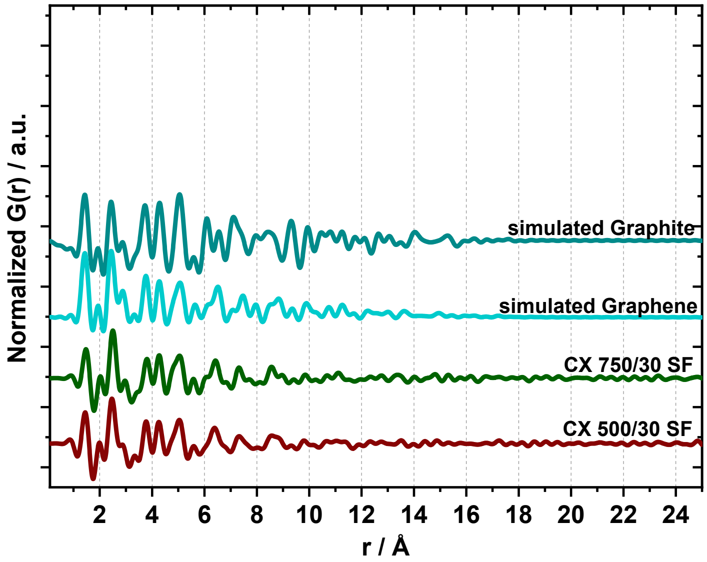

Fig. S5. Experimental PDFs of differently synthesized CX 750/30 (right) and compared with simulated PDFs of graphite and monolayer graphene..

5. X-ray photoelectron spectroscopy (XPS)

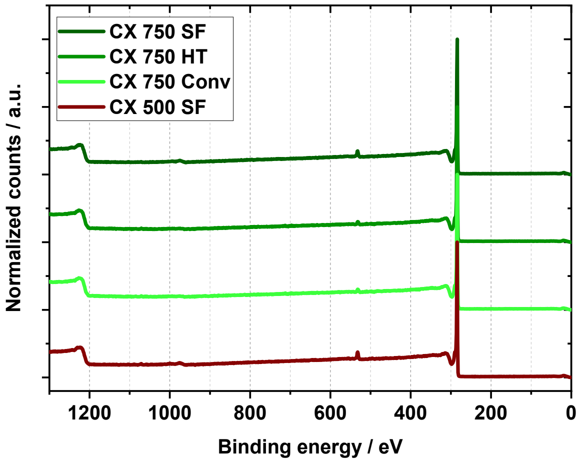

Fig. S6. C 1s spectra of differently synthesized CX 750 and CX 500 SF.

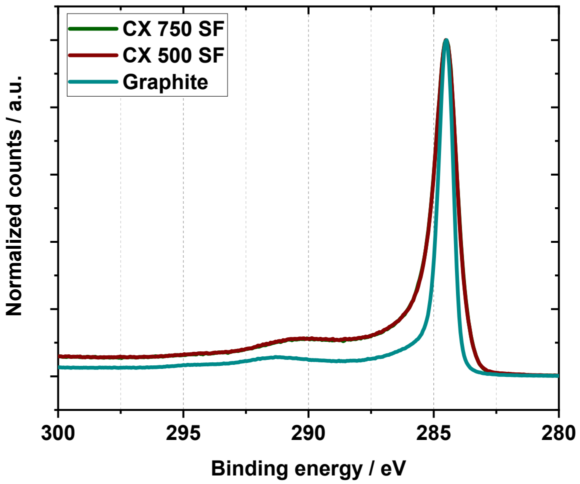

Fig. S7. High-resolution C 1s spectra of CX 500 SF, CX 750 SF (overlapping) and of graphite.

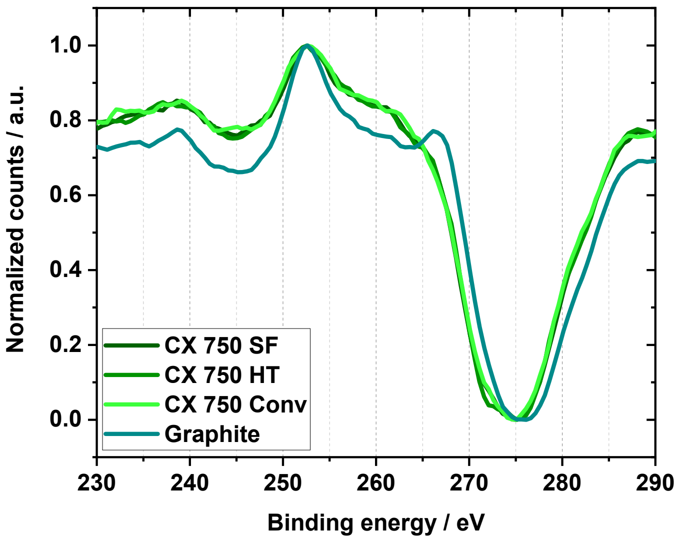

Fig. S8. The first derivatives of the Auger spectra of CX 750 series and graphite. The difference in binding energies between the maximum and the minimum is denoted as the D-parameter and is indicative for the ratio of sp2 to sp3 hybridization of carbon species.

Complementary Information (not mentioned in the main text)

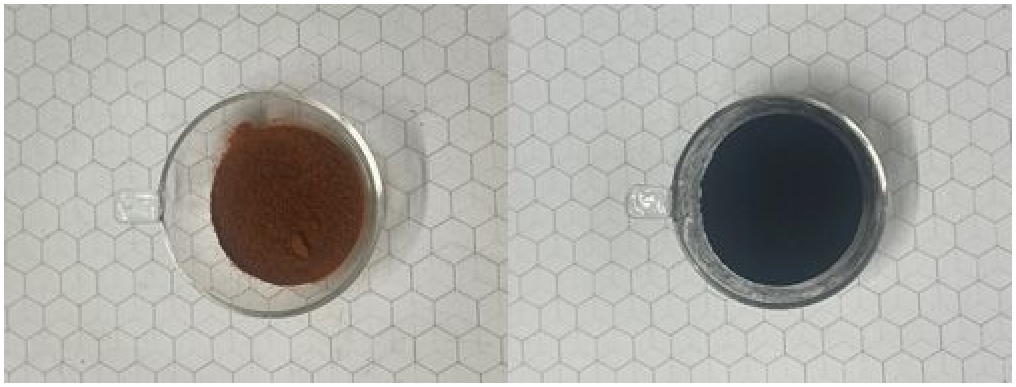

Fig. S9. Photographs of the crushed RF gel (left) and the carbon xerogel (right) obtained via the superfast synthesis route.

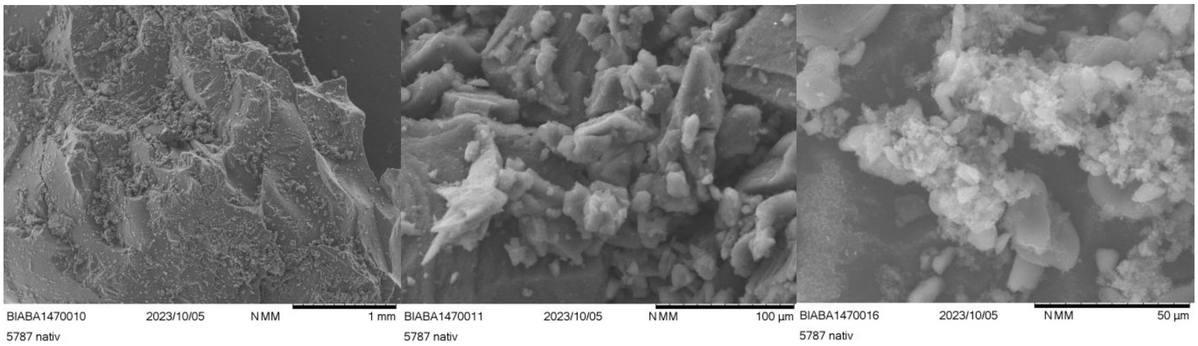

Fig. S10. SEM images of the carbon xerogel obtained via the superfast synthesis route.

As nitrogen molecules have a quadrupolar momentum, sorption data on porous carbon materials measured with nitrogen as the adsorbate may result in incorrect data with respect to pore sizes/volumes, especially if the surfaces of the given materials is highly polar. Argon is therefore recommended as a non-polar adsorbate resulting in unbiased data. For checking the validity of our sorption data if using nitrogen as adsorbate, isotherms were measured with nitrogen at 77 K and with argon at 87 K (Fig. S11). The data were measured on a Micromeritics 3Flex instrument, data evaluation was performed using the Quantachrome NovaWin software package, using the NLDFT kernels for adsorption of nitrogen/argon in carbon slit pores at 77/87 K. The comparison of the data obtained from both isotherms shows that the data with respect to micropore volume (V_{microp}), total pore volume (V_{tot}), and mean mesopore size (D_{mesop}) do not differ significantly as illustrated in Fig. S12. As no significant difference was observed using the advanced NLDFT data evaluation method, standard nitrogen adsorption data were used for data evaluation and comparison of the different carbon xerogels throughout the present work.

Fig. S11. Isotherms of sample CX 750 SF measured at 87 K for argon and at 77 K for nitrogen.

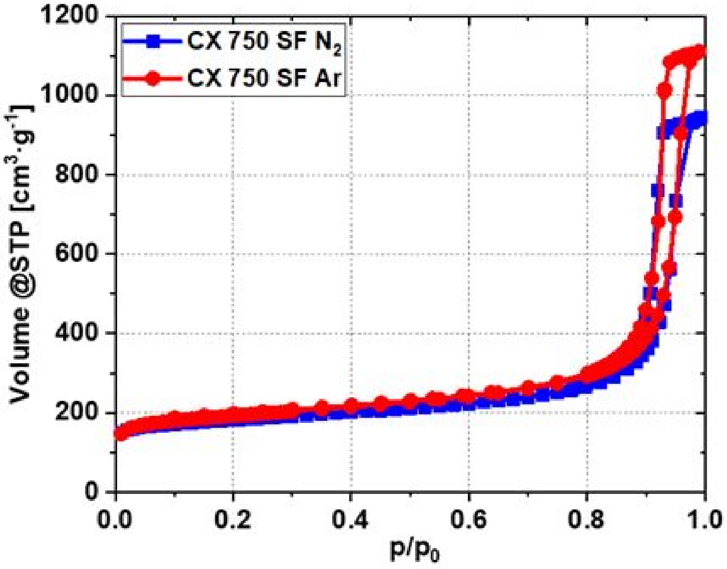

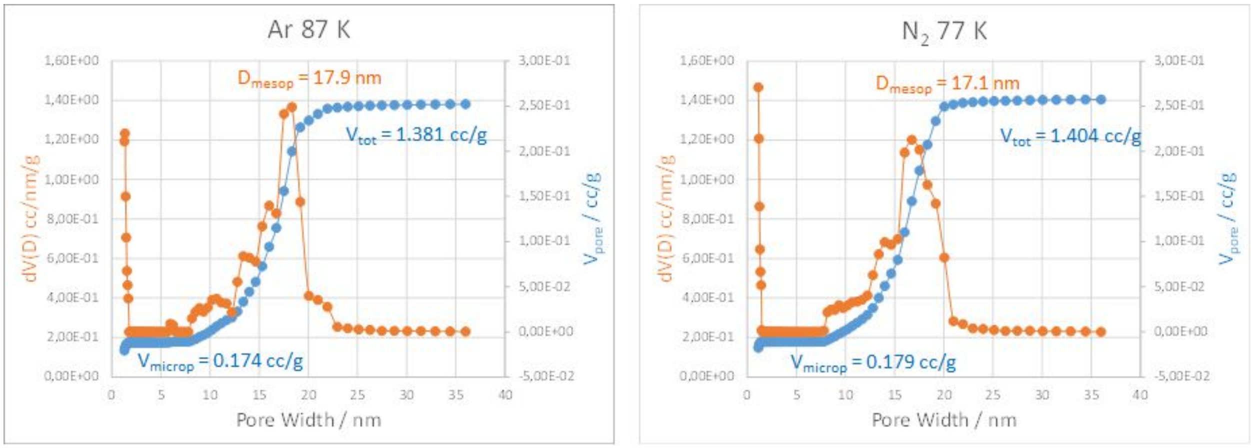

Fig. S12. Comparison of pore size distributions and cumulative pore volumes of CX 750 SF as obtained from the isotherms shown in Fig. S11. Data evaluation was performed here using th NovaWin software package provided by Quantachrome, using the NLDFT kernels for N2/Ar adsorption within carbon slit pores at 77/87 K.

Literature Supplementary Information

S1. Brunauer, S.; Emmett, P. H.; Teller, E., Adsorption of Gases in Multimolecular Layers. *Journal of the American Chemical Society* **1938,** *60* (2), 309-319.

S2. Rouquerol, J.; Llewellyn, P.; Rouquerol, F., Is the BET equation applicable to microporous adsorbents? *Characterization of Porous Solids VII - Studies in Surface Science and Catalysis 160*.

S3. Thommes, M.; Kaneko, K.; Neimark, A. V.; Olivier, J. P.; Rodriguez-Reinoso, F.; Rouquerol, J.; Sing, K. S. W., Physisorption of gases, with special reference to the evaluation of surface area and pore size distribution (IUPAC Technical Report) %J Pure and Applied Chemistry. *Pure and Applied Chemistry* **2015,** *87* (9-10), 1051-1069.

S4. Harkins, W. D.; Jura, G., Surfaces of Solids. XIII. A Vapor Adsorption Method for the Determination of the Area of a Solid without the Assumption of a Molecular Area, and the Areas Occupied by Nitrogen and Other Molecules on the Surface of a Solid. *Journal of the American Chemical Society* **1944,** *66* (8), 1366-1373.

S5. Hudec, P.; Smiešková, A.; idek, Z.; Schneider, P.; Ŝolcová, O., Determination of microporous structure of zeolites by t-plot method—State-of-the-art. In *Studies in Surface Science and Catalysis*, Aiello, R.; Giordano, G.; Testa, F., Eds. Elsevier: 2002; Vol. 142, pp 1587-1594.

S6. Allen, A. J.; Zhang, F.; Kline, R. J.; Guthrie, W. F.; Ilavsky, J., NIST Standard Reference Material 3600: Absolute Intensity Calibration Standard for Small-Angle X-ray Scattering. *Journal of Applied Crystallography* **2017,** *50* (2), 462-474.

S7. Guinier, A., La diffraction des rayons X aux très petits angles : application à l'étude de phénomènes ultramicroscopiques. *Ann. Phys.* **1939,** *11* (12), 161-237.

S8. Glatter, O. K. O., *Small angle x-ray scattering*. Academic Press: London; New York, 1982.

S9. Schnablegger, H.; Yashveer, S., The SAXS guide. Getting acquainted with the principles (Anton Paar GmbH). **2013**.

S10. Juhas, P.; Davis, T.; Farrow, C. L.; Billinge, S. J. L., PDFgetX3: a rapid and highly automatable program for processing powder diffraction data into total scattering pair distribution functions. *Journal of Applied Crystallography* **2013,** *46* (2), 560-566.

S11. Farrow, C. L.; Juhas, P.; Liu, J. W.; Bryndin, D.; Božin, E. S.; Bloch, J.; Proffen, T.; Billinge, S. J. L., PDFfit2 and PDFgui: computer programs for studying nanostructure in crystals. *Journal of Physics: Condensed Matter* **2007,** *19* (33), 335219.

S12. Sadezky, A.; Muckenhuber, H.; Grothe, H.; Niessner, R.; Pöschl, U., Raman microspectroscopy of soot and related carbonaceous materials: Spectral analysis and structural information. *Carbon* **2005,** *43* (8), 1731-1742.

S13. Fairley, N.; Fernandez, V.; Richard-Plouet, M.; Guillot-Deudon, C.; Walton, J.; Smith, E.; Flahaut, D.; Greiner, M.; Biesinger, M.; Tougaard, S.; Morgan, D.; Baltrusaitis, J., Systematic and collaborative approach to problem solving using X-ray photoelectron spectroscopy. *Applied Surface Science Advances* **2021,** *5*, 100112.

S14. Morgan, D. J., Comments on the XPS Analysis of Carbon Materials. *Journal of Carbon Research* **2021,** *7* (3), 51.
